# Supplementary material for: Combined metabolic activators therapy ameliorates liver fat in nonalcoholic fatty liver disease patients
Source: Mol Syst Biol. 2021 Oct 25;17(10):e10459. doi: 10.15252/msb.202110459 (PMC8724764; doi:10.15252/msb.202110459)
Supplement: Supplementary file 2 — Expanded View Figures PDF [file MSB-17-e10459-s010.pdf]

Expanded View Figures

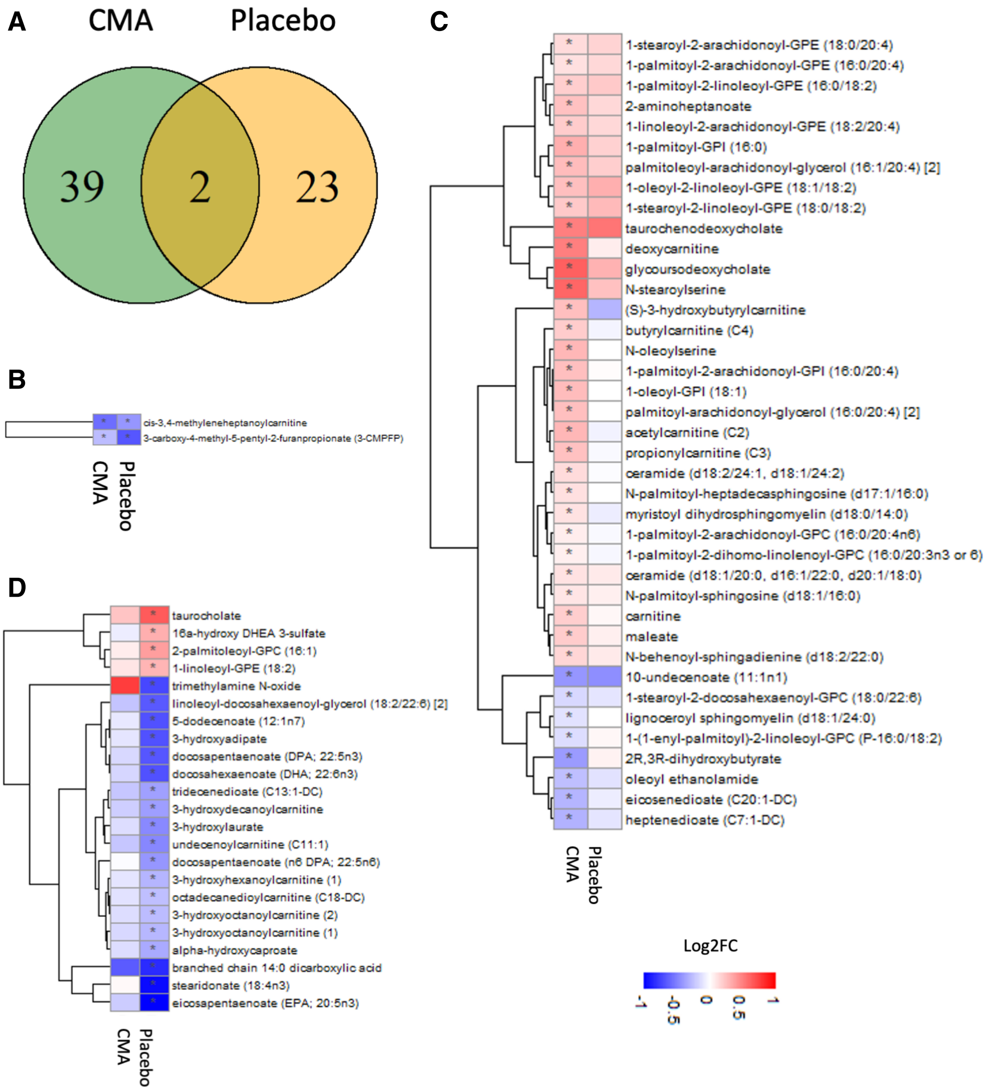

Figure EV1. CMA effects the plasma level of lipids.

Plasma level of lipids that are significantly different on Days 70 versus Day 0 in the CMA and placebo groups after weight loss adjustments.

A Venn diagram representing the number of identified lipids that are significantly different on Days 70 versus Day 0 in the CMA and placebo groups. The intersection represents lipids that are significantly different in both groups.

B–D Association between the plasma level of significantly different lipids (B) in both groups ($n = 2$); (C) only in CMA group ($n = 39$) and (D) only in placebo group ($n = 23$) on Day 70 versus Day 0. Heatmap shows log2FC-based alterations in the lipids. Asterisks indicate statistical significance based on paired Student's t-test. $P < 0.05$. Log2FC, log2(fold change).

© 2021 The Authors

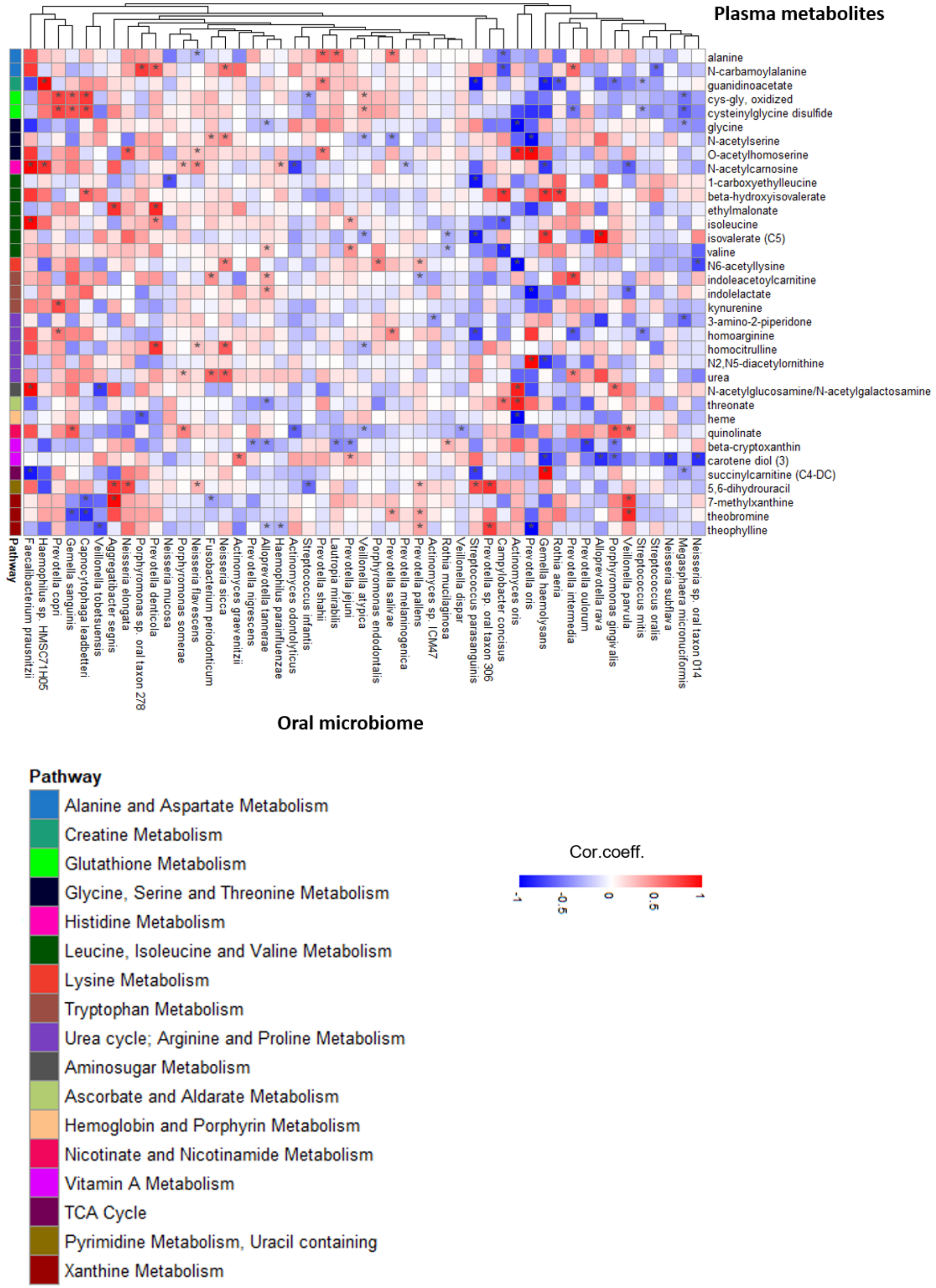

Figure EV2. Associations between the abundance of species in oral microbiome and the plasma level of metabolites (other than lipids).

Heatmaps show the correlations between the plasma levels of metabolites (other than lipids) and the abundance of the species in oral microbiome. Asterisks indicate statistical significance based on Spearman correlation analysis. $P < 0.05$; Cor. Coeff: correlation coefficient.

© 2021 The Authors

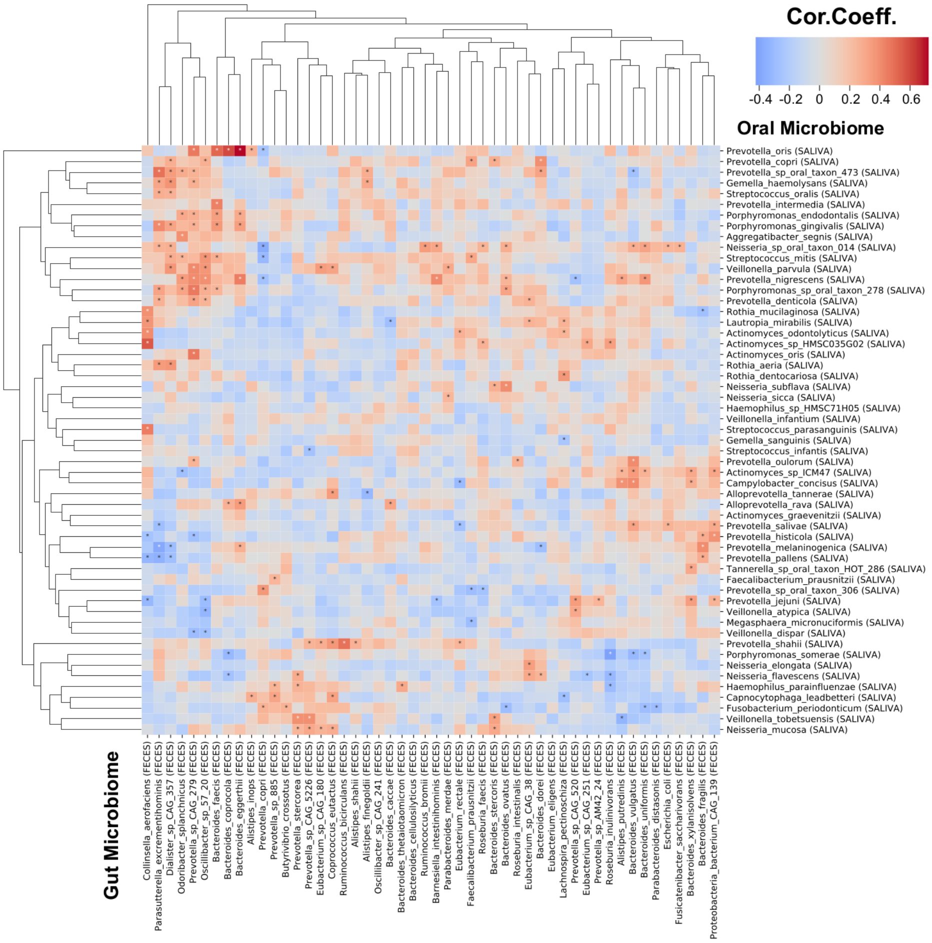

Figure EV3. Associations between the abundance of species in oral microbiome and gut microbiome.

Heatmap shows the correlations between the abundance of the species in oral microbiome and gut microbiome. Asterisks indicate statistical significance based on Spearman correlation analysis. $P < 0.05$; Cor. Coeff: correlation coefficient.

© 2021 The Authors

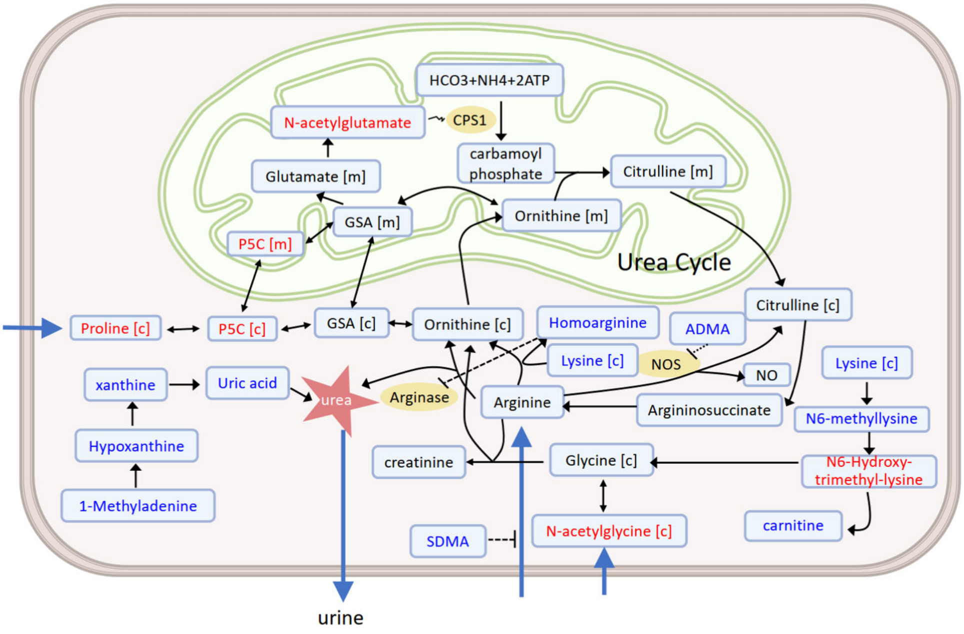

Figure EV4. CMA decreases uric acid plasma levels.

The uric acid level is significantly decreased on Day 70 versus Day 0 in the CMA group. Changes in metabolites or intermediates in key metabolic reactions are highlighted: Red and blue indicate the metabolite that was significantly increased and decreased, respectively, in the CMA group versus the placebo group on Day 70. Letters in brackets indicate subcellular compartments: [c], cytosol; [m] mitochondria. Arrows and dashed arrow link direct and indirect interactions, respectively. Black dashed line ending in a bar indicates a reaction inhibited by a certain metabolite. For example, asymmetric dimethylarginine (ADMA) is a potent inhibitor of nitric oxide synthase (NOS), whereas symmetric dimethylarginine (SDMA) inhibits nitric oxide (NO) production by inhibiting cellular uptake of arginine. CPS1 is regulated allosterically by N-acetyl glutamate, indicated by paired wavy lines. P5C, 1-pyrroline-5-carboxylate; GSA, L-glutamate 5-semialdehyde; CPS1, carbamoyl phosphate synthase 1. The blue arrows indicated that N-acetylglycine and proline are imported from extracellular, and the urea is secreted.

 © 2021 The Authors
