# Supplementary material for: Combined metabolic activators therapy ameliorates liver fat in nonalcoholic fatty liver disease patients
Source: Mol Syst Biol. 2021 Oct 25;17(10):e10459. doi: 10.15252/msb.202110459 (PMC8724764; doi:10.15252/msb.202110459)
Supplement: Supplementary file 21 [file MSB-17-e10459-s003.pdf]

molecular systems biology

Combined Metabolic Activators Therapy Ameliorates Liver Fat in Nonalcoholic Fatty Liver Disease Patients

Mujdat Zeybel, Ozlem Altay, Muhammad Arif, Xiangyu Li, hong Yang, Claudia Fredolini, Murat Akyildiz, Burcin Saglam, Mehmet Gokhan Gonenli, Dilek Ural, Woonghee Kim, Jochen Schwenk, Cheng Zhang, Saeed Shoaie, Jens Nielsen, Mathias Uhlén, Jan Boren, and Adil Mardinoglu
DOI: 10.15252/msb.202110459

Corresponding author(s): Adil Mardinoglu (adilm@scilifelab.se) , Jan Boren (jan.boren@wlab.gu.se)

Review Timeline:

Submission Date:	19th May 21
Editorial Decision:	19th Jul 21
Revision Received:	12th Aug 21
Editorial Decision:	10th Sep 21
Revision Received:	16th Sep 21
Accepted:	29th Sep 21

Editor: Jingyi Hou

Transaction Report:

(Note: With the exception of the correction of typographical or spelling errors that could be a source of ambiguity, letters and reports are not edited. Depending on transfer agreements, referee reports obtained elsewhere may or may not be included in this compilation. Referee reports are anonymous unless the Referee chooses to sign their reports.)

Thank you for submitting your work to Molecular Systems Biology. We have now heard back from two of the three reviewers who agreed to evaluate your manuscript. Unfortunately, after a series of reminders, we did not manage to obtain a report from Reviewer #4. In the interest of time, and since the recommendations of the other two reviewers are quite similar, I prefer to make a decision now rather than further delaying the process. As you will see from the reports below, the reviewers acknowledge the potential interest of the study. They raise however a series of concerns, which we would ask you to address in a major revision.

Since the reviewers' recommendations are rather clear, there is no need to reiterate all the points listed below. Some of the key issues that would need to be addressed are the following:

- The gender and age of the participants need to be considered in the data analysis.
- Reviewer #3 is concerned about the smaller number of controls compared to the study group, which needs to be justified, and limitations in this regard need to be discussed.
- In light of the concerns of Reviewer # 3, we would ask you to edit the manuscript to make sure that the microbiome part is sufficiently clear and easily accessible to the general audience of Molecular Systems Biology.

All other issues raised by the reviewers need to be satisfactorily addressed as well. As you may already know, our editorial policy allows in principle a single round of major revision and it is therefore essential to provide responses to the reviewers' comments that are as complete as possible.

On a more editorial level, we would ask you to address the following issues:

Reviewer #1:

In the present work, Zeybel et al. have studied the efficacy of combined metabolic activators (CMA) in nonalcoholic fatty liver disease (NAFLD) patients in a placebo-controlled study. The results of plasma metabolites, proteins and gut and oral microbiome are compared between CMA and placebo groups at Day 14 and 70. The study has some interesting findings and potential for treatment strategy in NAFLD patients. Some of the concerns related to the methods and results in the study are:

Major points:
1. Of the 31 individuals who participated in this study, 24 were male and 7 were female. Did the authors observe any sex-specific changes in the CMA and placebo group? Some of the metabolites reported in this study, such as those that are part of tryptophan metabolism, are affected by sex of the individual. How do the authors justify that the results observed in the CMA and placebo group is not masked by the male population in this study?

2. For the statistical analysis, the authors have adjusted for weight loss in the linear model. Why wasn't sex and age considered for adjusting along with the weight loss? Both sex and age does have a major influence on the metabolic changes observed in the individuals. It will be good to show how the trend changes when the calculations are adjusted for age, sex and weight loss.

3. For the intervention study, why were the patients in treatment group given a single dose of CMA for 2 weeks and then two doses for the next 56 days? Did increasing the dose after 14 days have an influence in the results observed for patients who took CMA compared to the placebo group?

4. For the inflammatory protein markers measured in this study, can the authors comment whether these observed proteins (CD8A, CSF-1, CCL23, FGF-21, and oncostatin-M (OSM)) are associated with increasing pro- or anti-inflammation response in the treated and placebo groups?

5. Did CMA treatment have an influence on the bile acid levels in the individuals? Considering bile acids have an important role in the progression of NAFLD, it is worthwhile to focus on the primary and secondary bile acids in the treatment and placebo group. Dataset S6 has some primary bile acids that are significant in the treated group.

6. Are the authors planning a follow-up study of these individuals beyond 70 days of treatment? Does CMA treatment have a permanent effect on the oxidation of fats and mitochondrial function? As the treatment was stopped after 70 days, did the treated individuals revert back to the original state?

Minor points:
1. The authors should mention in the methods section when was the blood drawn done (time of the day) and whether it was fasted blood measurements or not. Also, when was the stool sample collected for these individuals? These details are currently lacking in the manuscript.

Reviewer #3:

Zeybel et al., describe how Combined metabolic activators (CMA) act as reducers of liver fat in NAFLD patients. The paper is interesting with potential non-harmful impact for treatment of NAFLD in the future. It drives from previous studies from the Mardinglu group. Here, the authors performed a small clinical study with patients treated with a combination of CMA for 70 days. They provide a thorough analysis, particularly of metabolome and microbiome, in the treated and the control groups. The acquisition of data is appropriate, also the data analysis. The paper is a bit lengthy in the microbiome part since there are no clear conclusions. The paper is not always easy to follow. Some major facts are missing in the main part and might have been overlooked in the comprehensive supplementary files. Some figures that would facilitate the reader to follow the story should be produced.
Major comments:
1. A drawback of the study is its small size and the number of controls smaller compared to the study group (2 : 1). Good study design requires the control group to be at least of the same size or larger compared to the treated group. The authors should justify why they believe that the 2 : 1 ratio (treated : control) would be sufficient for this study.

2. It is not clear how the patients were monitored during the 70-day protocol - I understood they were living at home and returning to the clinics for routine investigations connected to the study. This is a crucial piece of information that needs to be visible. If patients were treated in an outpatient clinic setting, this can be the reason for additional variation of the microbiome.

3. I could not find the information how were patients dispersed by gender. Were they only males? If they were of both sexes it should be explained that both gender groups were combined in statistical analyses.

4. CMA: The composition should be visible in the main paper. If it is a part of IP protection it has to be disclosed.

5. From the biochemical standpoint there is a statistically significant difference between the control and treated groups in

multiple metabolites, some of them not described in detail in the paper. Could the authors explain why i. e. the changes in blood lipid composition was not studies deeper?

6. The authors disclose that the fat content of the liver was evaluated by the MRI method and no biopsies were taken. A clinician should judge whether the MRI fat data from table 1 are sufficient to declare in the manuscript title that CMAs reduce the liver fat. Certainly, the patients have lost weight. Is this only the effect of CMA? another clinical trial in biopsy proven NASH is needed to delineate the effects of CMA on hepatic injury and inflammation.

7. Safety issues: it is not clear whether the side effects described are linked to CMA or are certainly not linked to the treatment.

8. Certain weight loss combinations of drugs can have also detrimental effect in human health. Authors should better explain why lowering uric acid and creatinine is certainly beneficial for patients.

9. The microbiome part can be in the present form understood only by microbiome specialists and not for the general biochemistry audience. What was expected and what has been found?

10. The underlying molecular mechanism associated with the the reduced hepatic fat and inflammation in NAFLD patients and key player sin host-microbiome interactions should be underlined better and presented also in a graphical manner.

Detailed point-by-point response to the reviewer comments

The authors would like to thank the reviewers for their time and expert comments and the editor for providing us with the opportunity to resubmit our manuscript to the journal following corrections and further changes as requested. We have re-written the text to address the points raised by the reviewers. Our answers to reviewers' questions can be found in blue in the text below. We hope that the reviewers and editors agree that the manuscript has been markedly improved as a result of these changes and that it is now suitable for publication.

To support our findings in our study, we also added results from our recently completed clinical trials related to COVID-19, Alzheimer's Disease and Parkinson's Disease.

'The cellular levels of NAD+ and GSH are fundamental factors related to the metabolic conditions and the risk for mortality (Miller et al, 2020; Silvagno et al, 2020). Depleted GSH can be restored by administration of NAC, serine and glycine which can be synthesized through the interconversion of serine. (Sim et al, 2014). Two other components of CMA including carnitine and NR, which stimulate the transfer of fatty acids from the cytosol to mitochondria, are also depleted in liver diseases (Bieganowski & Brenner, 2004; Hagen et al, 1998; Salic et al, 2019; Savic et al, 2020; Trammell et al, 2016). Overall improvement of clinical homeostasis reduces oxidative injury and improve bioenergetics; thereby is more likely to have successful outcomes (Cummings et al, 2020). According to this, we recently demonstrated the efficacy of CMA for different metabolic conditions in a short-term (14 days) Phase 2/3COVID-19 clinical trials (Altay et al, 2021) and two longer-term (12 weeks) Phase 2 Alzheimer's Disease (AD) (Yulug et al, 2021a) and Phase 2 Parkinson's Disease (PD) (Yulug et al, 2021b) clinical trials. Notably, we observed the administration of CMA decreased the levels of ALT and creatinine in all short term and longer-term clinical trials as well as it decreased the level of uric acid in longer-term AD and PD Phase 2 clinical trials.'

Reviewer #1:
In the present work, Zeybel et al. have studied the efficacy of combined metabolic activators (CMA) in nonalcoholic fatty liver disease (NAFLD) patients in a placebo-controlled study. The results of plasma metabolites, proteins and gut and oral microbiome are compared between CMA and placebo groups at Day 14 and 70. The study has some interesting findings and potential for treatment strategy in NAFLD patients. Some of the concerns related to the methods and results in the study are:

Major points:
1. Of the 31 individuals who participated in this study, 24 were male and 7 were female. Did the authors observe any sex-specific changes in the CMA and placebo group? Some of the metabolites reported in this study, such as those that are part of tryptophan metabolism, are

affected by sex of the individual. How do the authors justify that the results observed in the CMA and placebo group is not masked by the male population in this study?.

Response:

We appreciate the reviewer's comment regarding overrepresentation of male participants in the study. Generally speaking, NAFLD prevalence is at least two times higher in men than women[1], however, male subjects were even more predominant in our study. We believe that this was at least partly due to the exclusion of women who are planning pregnancy, breastfeeding, and reluctance to use contraception during the study. Although we attempted to analyse sex-specific changes in metabolite levels, the sample size for female subjects were rather small (3 in CMA group and 4 in placebo), statistical analysis would have been generated with a low statistical power. This is the limitation of this study that we have now highlighted in the manuscript as shown below. We have taken this comment on board and we will ensure the fair female/male recruitment in the design of the phase 2b/phase 3 study.

'Moreover, only 7 female subjects (3 from CMA and 4 from placebo group) remained in the sample pool, which hinder the possibility to perform sex-specific analysis.'

[1]Lonardo, A., Nascimbeni, F., Ballestri, S., Fairweather, D., Win, S., Than, T.A., Abdelmalek, M.F. and Suzuki, A. (2019), Sex Differences in Nonalcoholic Fatty Liver Disease: State of the Art and Identification of Research Gaps. Hepatology, 70: 1457-1469. https://doi.org/10.1002/hep.30626

2. For the statistical analysis, the authors have adjusted for weight loss in the linear model. Why wasn't sex and age considered for adjusting along with the weight loss? Both sex and age does have a major influence on the metabolic changes observed in the individuals. It will be good to show how the trend changes when the calculations are adjusted for age, sex and weight loss.

Response:

We very much appreciate this point. We realized that we missed an important explanation in the statistical analysis section, that we performed this analysis in a pairwise manner. That was done to avoid the effect of individual variations, since we were comparing each subject to themself in different timepoints. So with this paired analysis, adding sex and age in the model will not make any difference in the results since these variables were constant for the same subject, unlike weight variables that changed between visits for each subject. We have added the clarification about the paired analysis in the statistical analysis section.

'The calculation was done by pairing each subject from different visits and adjusted for weight loss in a linear model using the limma package in R (v4.0.2)'

3. For the intervention study, why were the patients in the treatment group given a single dose of CMA for 2 weeks and then two doses for the next 56 days? Did increasing the dose after 14 days

have an influence in the results observed for patients who took CMA compared to the placebo group?

Response:

We determined the safe dose by considering the findings in the non-clinical animal and phase-1 human studies as well as the existing literature of the constituents of the CMA[1,2]. In order to limit and monitor the potential adverse reactions in NAFLD patients (due to the potential differences in target distribution and pharmacokinetics of the drug between healthy volunteers and patients), we used a lower starting dose that is expected to have a minimal pharmacological effect and is safer to use. Later, we increased the doses (twice a day) and continued screening the patients during the study.

[1]C. Zhang, E. Bjornson, M. Arif, A. Tebani, A. Lovric, R. Benfeitas, M. Ozcan, K. Juszczak, W. Kim, J. T. Kim, G. Bidkhori, M. Ståhlman, P.-O. Bergh, M. Adiels, H. Turkez, M.-R. Taskinen, J. Bosley, H.-U. Marschall, J. Nielsen, M. Uhlén, J. Borén, A. Mardinoglu, The acute effect of metabolic cofactor supplementation: a potential therapeutic strategy against non-alcoholic fatty liver disease. Mol Syst Biol 16, e9495-e9495 (2020).

[2]A. Mardinoglu, D. Ural, M. Zeybel, H. H. Yuksel, M. Uhlén, J. Borén, The Potential Use of Metabolic Cofactors in Treatment of NAFLD. Nutrients 11, 1578 (2019).

4. For the inflammatory protein markers measured in this study, can the authors comment whether these observed proteins (CD8A, CSF-1, CCL23, FGF-21, and oncostatin-M (OSM)) are associated with increasing pro- or anti-inflammation response in the treated and placebo groups?

Response:

We appreciate this comment. Generally speaking, these proteins are pro-inflammatory but FGF-21 might have anti-inflammatory properties in this context. A long acting FGF-21 analogue attenuated hepatic inflammation in animal models of liver disease[1]. These proteins are induced by a variety of environmental or metabolic stimuli, play crucial roles in the response to these challenges. As example: 1) CD8 has been well known to be expressed in cytotoxic T cells to recognize and kill infected cells. CD8, usually composed of one CD8α and one CD8β chain[2]; 2) CSF-1 is a key differentiation, growth and survival factor for monocytes/macrophages and osteoclasts[3]; 3) CCL23 has been reported to be expressed in activated monocytes and dendritic cells and is a biomarker for some inflammatory diseases[4]; 4) Fibroblast growth factor 21 (FGF21) levels are increased by fasting, fibrate treatment, obesity, and type 2 diabetes[5]; 5) Oncostatin M is a member of the IL-6 family of cytokines that induces a key endothelial cell function, initiation of the inflammatory response[6].

[1]Keinicke, Helle, Gao Sun, Caroline M Junker Mentzel, Merete Fredholm, Linu Mary John, Birgitte Andersen, Kirsten Raun, and Marina Kjaergaard. " FGF21 regulates hepatic metabolic pathways to improve steatosis and inflammation", Endocrine Connections 9, 8 (2020): 755-768, accessed Aug 10, 2021, https://doi.org/10.1530/EC-20-0152

[2]Matsuzaki, J. et al. A rare population of tumor antigen-specific CD4+CD8+ double-positive αβ T lymphocytes uniquely provide CD8-independent TCR genes for engineering therapeutic T cells. Journal for ImmunoTherapy of Cancer 7, 7, doi:10.1186/s40425-018-0467-y (2019).

[3]Garcia, S. et al. Colony-stimulating factor (CSF) 1 receptor blockade reduces inflammation in human and murine models of rheumatoid arthritis. Arthritis Research & Therapy 18, 75, doi:10.1186/s13075-016-0973-6 (2016).

[4]Poposki, J. A. et al. Increased expression of the chemokine CCL23 in eosinophilic chronic rhinosinusitis with nasal polyps. J Allergy Clin Immunol 128, 73-81.e74, doi:10.1016/j.jaci.2011.03.017 (2011).
[5]Kim, K. H. & Lee, M.-S. FGF21 as a mediator of adaptive responses to stress and metabolic benefits of anti-diabetic drugs. Journal of Endocrinology 226, R1-R16, doi:10.1530/JOE-15-0160 (2015).
[6]Modur, V. et al. Oncostatin M is a proinflammatory mediator. In vivo effects correlate with endothelial cell expression of inflammatory cytokines and adhesion molecules. J Clin Invest 100, 158-168, doi:10.1172/JCI119508 (1997).

5. Did CMA treatment have an influence on the bile acid levels in the individuals? Considering bile acids have an important role in the progression of NAFLD, it is worthwhile to focus on the primary and secondary bile acids in the treatment and placebo group. Dataset S6 has some primary bile acids that are significant in the treated group.

Response:
In total, we measured 32 metabolites belonging to bile acid metabolism. As shown in the summary table of Dataset S6 below, only 2 of them (taurochenodeoxycholate and glycoursodeoxycholate) were significantly increased between Day 70 vs Day 0 in CMA group than placebo. There are also a few significant metabolites both in placebo and drug groups on Day 14 and Day 70 (yellow marked). However, we concluded that these findings require further validation in a larger clinical trial as well as a longer treatment period.

SUB PATH WAY	Metabolite Name	Day 14 vs Day 0				Day 70 vs Day 0			
		Placebo		Treated		Placebo		Treated	
		Log2FC	P-Value	Log2FC	P-Value	Log2FC	P-Value	Log2FC	P-Value
Primary Bile Acid Metabolism	chenodeoxycholate	0,086	0,627	-0,400	0,144	0,181	0,310	-0,260	0,272
	cholate	0,479	0,375	-0,104	0,828	0,108	0,844	-0,462	0,278
	cholic acid glucuronide	-0,054		0,085		-0,201		-0,346	
	glyco-beta-muricholate	0,411	0,369	0,286		0,681	0,197	0,195	
	glycochenodeoxycholate	0,333	0,338	0,148	0,573	0,601	0,101	0,446	0,060
	glycochenodeoxycholate 3-sulfate	-0,298	0,091	0,346	**0,042**	0,294	0,103	0,225	0,127
	glycochenodeoxycholate glucuronide (1)	-0,031	0,883	-0,026	0,877	0,039	0,860	0,196	0,191

	glycocholate	0,150	0,590	0,524	**0,039**	0,370	0,203	0,345	0,117
	taurochenodeoxycholate	0,202	0,510	0,729	**0,010**	0,551	0,090	0,516	**0,034**
	taurocholate	0,417	0,107	0,605	0,060	0,653	**0,019**	0,249	0,368
Secondary Bile Acid Metabolism	3b-hydroxy-5-cholenoic acid	-0,204		-0,051		-0,225		-0,250	
	7-ketodeoxycholate	-0,053		-0,124		0,193		-0,135	
	deoxycholate	-0,077		-0,350		-0,591		-0,298	
	deoxycholic acid 12-sulfate*	-0,554	0,149	-0,240	0,237	-0,743	0,085	-0,252	0,160
	deoxycholic acid glucuronide	0,082	0,698	-0,150	0,290	-0,137	0,538	-0,108	0,384
	glycocholenate sulfate*	-0,009	0,906	-0,071	0,400	0,025	0,739	-0,009	0,898
	glycodeoxycholate	-0,215		0,223		-0,232		0,394	
	glycodeoxycholate 3-sulfate	-0,458	0,126	0,175	0,337	0,304	0,313	0,198	0,220
	glycohyocholate	0,770		0,366		0,520		0,297	
	glycolithocholate	0,265		-0,531		0,488		-0,012	
	glycolithocholate sulfate*	-0,899	**0,007**	0,126	0,434	-0,439	0,167	0,195	0,173
	glycoursodeoxycholate	0,376	0,169	0,183	0,460	0,316	0,257	0,635	**0,006**
	glycoursodeoxycholic acid sulfate (1)	0,279	0,243	0,020		-0,009	0,971	0,542	
	hyocholate	0,090	0,472	0,044	0,767	-0,118	0,364	-0,039	0,767
	isoursodeoxycholate	0,230	0,438	-0,097	0,662	-0,623	0,050	0,297	0,134

	lithocholate sulfate (1)	-0,203	0,602	-0,141	0,568	-0,373	0,363	0,149	0,496
	taurochenodeoxycholic acid 3-sulfate	-0,219		0,330	0,138	0,545		0,146	0,437
	taurocholenate sulfate*	-0,175	0,473	0,004	0,980	0,074	0,765	0,159	0,252
	taurodeoxycholate	0,020		1,156		0,102		0,634	
	taurodeoxycholic acid 3-sulfate	0,104	0,744	0,248		0,392	0,258	0,381	
	taurolithocholate 3-sulfate	-0,896	**0,018**	0,311	0,154	-0,187	0,603	0,248	0,196
	ursodeoxycholate	-0,061	0,868	-0,520	0,068	-0,325	0,393	-0,139	0,570

6. Are the authors planning a follow-up study of these individuals beyond 70 days of treatment? Does CMA treatment have a permanent effect on the oxidation of fats and mitochondrial function? As the treatment was stopped after 70 days, did the treated individuals revert back to the original state?

Response:
Authors are currently planning a Phase 2b trial with a longer period of (36 weeks) treatment. We agree that adding an additional lag phase visit for investigating durability of treatment response is an excellent idea.

Minor points:
1. The authors should mention in the methods section when was the blood drawn done (time of the day) and whether it was fasted blood measurements or not. Also, when was the stool sample collected for these individuals? These details are currently lacking in the manuscript.

Response:
We thank the review for this comment. Fasting blood sampling was performed in the morning (8.00 am -10.00 am). Stool collection kits were provided to the patients and advised to collect stool samples in the last 24 hours of the visit. We have now clarified this in the Method and Extended View Appendix section.

Reviewer #3:

Zeybel et al., describe how Combined metabolic activators (CMA) act as reducers of liver fat in NAFLD patients. The paper is interesting with potential non-harmful impact for treatment of NAFLD in the future. It drives from previous studies from the Mardinglu group. Here, the authors performed a small clinical study with patients treated with a combination of CMA for 70 days. They provide a thorough analysis, particularly of metabolome and microbiome, in the treated and the control groups. The acquisition of data is appropriate, also the data analysis. The paper is a bit lengthy in the microbiome part since there are no clear conclusions. The paper is not always easy to follow. Some major facts are missing in the main part and might have been overlooked in the comprehensive supplementary files. Some figures that would facilitate the reader to follow the story should be produced.

Major comments:

1. A drawback of the study is its small size and the number of controls smaller compared to the study group (2 : 1). Good study design requires the control group to be at least of the same size or larger compared to the treated group. The authors should justify why they believe that the 2:1 ratio (treated: control) would be sufficient for this study.

Response:

We agree with the reviewer that the sample size is not large. However, we followed patients longitudinally and performed comprehensive multi-omic analysis (proteomics, metabolomics and oral/gut metagenomics) and clinical evaluation at the same time point. With the encouraging results of this phase 2 study, we are planning to conduct phase 2b and 3 clinical trials with larger patient cohorts.

It is not uncommon to perform a 2:1 ratio of treatment and placebo in NAFLD clinical trials[1,2]. We also chose a 2:1 allocation and recruited more patients in the drug group for several reasons. These can be listed as: 1) gaining experience about the efficacy of the drug, 2) better evaluation of in-cohort changes between different time points, and 3) gathering more information about the side effects.

[1]Harrison, Stephen A. et al. Selonsertib for patients with bridging fibrosis or compensated cirrhosis due to NASH: Results from randomized phase III STELLAR trials. Journal of Hepatology, Volume 73, Issue 1, 26 - 39

[2]Harrison, Stephen A et al. Resmetirom (MGL-3196) for the treatment of non-alcoholic steatohepatitis: a multicentre, randomised, double-blind, placebo-controlled, phase 2 trial The Lancet, Volume 394, Issue 10213, 2012 - 2024

2. It is not clear how the patients were monitored during the 70-day protocol - I understood they were living at home and returning to the clinics for routine investigations connected to the study. This is a crucial piece of information that needs to be visible. If patients were treated in an outpatient clinic setting, this can be the reason for additional variation of the microbiome.

Response:

Some of the visits changed as “optional” on the study protocol due to COVID-19 pandemics and local restrictions. Clinical trial nurses performed weekly telephone visits and gathered crucial

clinical information including adverse events and assessed compliance to the medications. Patients (both treatment arms) were seen three times by dieticians and received advice for healthy diet. Despite these efforts and presence of a placebo arm we cannot rule out the effect of diet on microbiome.

3. I could not find the information how were patients dispersed by gender. Were they only males? If they were of both sexes it should be explained that both gender groups were combined in statistical analyses.

Response:
We thank the reviewer for this comment. We recruited 24 males and 7 females for this study. In general, NAFLD prevalence is at least two times higher in men than women[1]. The male subjects were more predominant in this study because we excluded the women who are planning pregnancy, breastfeeding, and reluctance to use contraception during the study according to the study protocol. We have now clarified this in the Methods section.

[1] Lonardo, A., Nascimbeni, F., Ballestri, S., Fairweather, D., Win, S., Than, T.A., Abdelmalek, M.F. and Suzuki, A. (2019), Sex Differences in Nonalcoholic Fatty Liver Disease: State of the Art and Identification of Research Gaps. Hepatology, 70: 1457-1469. https://doi.org/10.1002/hep.30626

4. CMA: The composition should be visible in the main paper. If it is a part of IP protection it has to be disclosed.

Response:
The composition of CMA has been provided in the main manuscript method section (under Randomization, Interventions, and Follow-up subtitle) as well as in the supplementary appendix. Please see the paragraph below.

"CMA treatment was given for 70 days after the initial diagnosis of high hepatic fat by MRI-PDFF. Patients in the treatment group took one dose of CMA (3.73 g L-carnitine tartrate, 1 g nicotinamide riboside, 12.35 g serine, and 2.55 g N-acetyl-l-cysteine) daily for the first 14 days (after dinner) and two doses daily for the next 56 days (after breakfast and dinner). Further information is provided in the Supplementary Appendix."

5. From the biochemical standpoint there is a statistically significant difference between the control and treated groups in multiple metabolites, some of them not described in detail in the paper. Could the authors explain why i. e. the changes in blood lipid composition was not studies deeper?

Response:
We thank the reviewer for the guidance. Although abnormal lipid metabolism is characteristic of NAFLD, this is often demonstrated at hepatic lipidomics level rather than plasma lipid changes.

Certain lipids mediate inflammatory pathways leading to oxidative stress and may contribute to disease progression. Interestingly, a few metabolites in fatty acid metabolism significantly altered, which 2R,3R-dihydroxybutyrate were decreased both on Day 14 and Day 70 in CMA group (p= 0,00007 and p= 0,0000006 respectively) while an increase of 3-hydroxybutyrate (BHBA) on Day 14 (p= 0,02). Landmark lipids of diacylglycerol and dihydroceramide levels were not altered throughout the study. We observed some alterations in phosphatidylcholine and phosphatidylethanolamine along with ceramide species; however, as we had not measured hepatic levels of these lipids, we concluded that the effect of CMA on hepatic lipid composition needs further hepatic lipid studies.

6. The authors disclose that the fat content of the liver was evaluated by the MRI method and no biopsies were taken. A clinician should judge whether the MRI fat data from table 1 are sufficient to declare in the manuscript title that CMAs reduce liver fat. Certainly, the patients have lost weight. Is this only the effect of CMA? Another clinical trial in biopsy proven NASH is needed to delineate the effects of CMA on hepatic injury and inflammation.

Response:
We thank the reviewer for the comment. Recent clinical studies demonstrated that Magnetic Resonance Imaging–derived proton density fat fraction (MRI-PDFF) can accurately measure hepatic fat content to assess treatment response in phase-2 NASH trials[1]. MRI-PDFF-based hepatic fat measurement shows excellent correlation with biopsy-based steatosis assessment[2]. However, we and regulatory authorities agree with the reviewer that there is a need to perform a biopsy proven Phase 3 NASH study to investigate the effect of supplementation on inflammation or steatohepatitis.

[1]Caussy, C., Reeder, S.B., Sirlin, C.B. and Loomba, R. (2018), Noninvasive, Quantitative Assessment of Liver Fat by MRI-PDFF as an Endpoint in NASH Trials. Hepatology, 68: 763-772. https://doi.org/10.1002/hep.29797

[2]Jayakumar S, Middleton MS, Lawitz EJ, Mantry PS, Caldwell SH, Arnold H, Mae Diehl A, Ghalib R, Elkhashab M, Abdelmalek MF, Kowdley KV, Stephen Djedjos C, Xu R, Han L, Mani Subramanian G, Myers RP, Goodman ZD, Afdhal NH, Charlton MR, Sirlin CB, Loomba R. Longitudinal correlations between MRE, MRI-PDFF, and liver histology in patients with non-alcoholic steatohepatitis: Analysis of data from a phase II trial of selonsertib. J Hepatol. 2019 Jan;70(1):133-141. doi: 10.1016/j.jhep.2018.09.024.

7. Safety issues: it is not clear whether the side effects described are linked to CMA or are certainly not linked to the treatment.

Response:
We do not have evidence to claim these adverse events are directly related to treatment. However, larger clinical trials with a longer treatment might provide evidence for the matter. Of note, we recently published a phase 2 and 3 clinical trial to evaluate the efficacy of CMA in COVID-19 patients. In a total of 397 participants, side effects were seen only in 4 (1%) of them[1]. All had a mild rash or skin changes and decided to complete the study without changing the dose.

[1]Altay, O., Arif, M., Li, X., Yang, H., Aydın, M., Alkurt, G., Kim, W., Akyol, D., Zhang, C., Dinler-Doganay, G., Turkez, H., Shoaie, S., Nielsen, J., Borén, J., Olmuscelik, O., Doganay, L., Uhlén, M., Mardinoglu, A., Combined Metabolic Activators Accelerates Recovery in Mild-to-Moderate COVID-19. Adv. Sci. 2021, 2101222. https://doi.org/10.1002/advs.202101222

8. Certain weight loss combinations of drugs can have also detrimental effect in human health. Authors should better explain why lowering uric acid and creatinine is certainly beneficial for patients.

Response:
Uric acid is a natural waste product from the digestion of foods that contain purines. Hyperuricemia is frequently associated wıth a number of clinical conditions including gout, hyperuricemic nephropathy and coronary artery disease[1]. Research has also shown a link between high uric acid levels and type 2 diabetes, high blood pressure, and fatty liver disease[2]. There is some evidence to suggest that patients with high creatinine levels in the blood may be more at risk for either developing kidney damage or for kidney damage that they already have getting worse[3]. However, there is limited data and larger studies are required to study the effect of creatinine lowering therapy on kidney protection.

[1] Lee, S.J., Oh, B.K. & Sung, KC. Uric acid and cardiometabolic diseases. Clin Hypertens 26, 13 (2020). https://doi.org/10.1186/s40885-020-00146-y

[2] Xiong Q, Liu J, Xu Y. Effects of Uric Acid on Diabetes Mellitus and Its Chronic Complications. Int J Endocrinol. 2019;2019:9691345. Published 2019 Oct 13. doi:10.1155/2019/9691345

[3] Mendelssohn DC, Barrett BJ, Brownscombe LM, Ethier J, Greenberg DE, Kanani SD, Levin A, Toffelmire EB. Elevated levels of serum creatinine: recommendations for management and referral. CMAJ. 1999 Aug 24;161(4):413-7. PMID: 10478168; PMCID: PMC1230545.

9. The microbiome part can be in the present form understood only by microbiome specialists and not for the general biochemistry audience. What was expected and what has been found?

Response:
We thank the reviewer for the comment. We extended and clarified the microbiome section in the discussion as below.
'The direct or indirect impact of the microbiome on NAFLD remains poorly understood, but numerous human and animal studies have confirmed the contribution of intestinal microbiota as a driver through the compositional alterations, changes in the levels of microbiota-derived metabolites, and loss of gut barrier integrity (Altay et al, 2019; Aron-Wisnewsky et al, 2020). Even though the inaccurate results have been obtained for patient-derived microbiome composition analyses, overgrowth of the Proteobacteria and Actinobacteria phyla as well as the increased Firmicutes/Bacteroidetes ratio have been shown as common markers of dysbiosis (Aron-Wisnewsky et al., 2020; Magne et al, 2020). In this study, we comprehensively characterized the gut and oral microbiome in NAFLD patients before and after administration of metabolic activators. We observed a significant decrease in species belonging to Proteobacteria, Actinobacteria, and Firmicutes in the CMA group after 70 days therapy. Additionally, the main end products of bacterial metabolism are short-chain fatty acids which especially butyrate is shown to reduce intestinal inflammation (Segain et al, 2000). In our study, well-known butyrate-

producing species are increased in the CMA group. Our results collectively indicated that CMA induced literature supported beneficial alterations such as positive correlation with the abundances of Faecalibacterium prausnitzii (Grabherr et al, 2019; Munukka et al, 2017), Roseburia faecis (Tamanai-Shacoori et al, 2017) or butyrate producers (Baxter et al, 2019; Boesmans et al, 2018). In addition to identifying the key species, defining the distinct signatures of the microbiome and liver-related clinical parameters in the well-defined patient cohort is valuable in searching for new biomarkers for NAFLD.'

Altay O, Nielsen J, Uhlen M, Boren J, Mardinoglu A (2019) Systems biology perspective for studying the gut microbiota in human physiology and liver diseases. EBioMedicine 49: 364-373

Aron-Wisnewsky J, Vigliotti C, Witjes J, Le P, Holleboom AG, Verheij J, Nieuwdorp M, Clément K (2020) Gut microbiota and human NAFLD: disentangling microbial signatures from metabolic disorders. Nature Reviews Gastroenterology & Hepatology 17: 279-297

Baxter NT, Schmidt AW, Venkataraman A, Kim KS, Waldron C, Schmidt TM (2019) Dynamics of Human Gut Microbiota and Short-Chain Fatty Acids in Response to Dietary Interventions with Three Fermentable Fibers. mBio 10: e02566-02518

Boesmans L, Valles-Colomer M, Wang J, Eeckhaut V, Falony G, Ducatelle R, Van Immerseel F, Raes J, Verbeke K (2018) Butyrate Producers as Potential Next-Generation Probiotics: Safety Assessment of the Administration of Butyricicoccus pullicaecorum to Healthy Volunteers. mSystems 3: e00094-00018

Grabherr F, Grander C, Effenberger M, Adolph TE, Tilg H (2019) Gut Dysfunction and Non-alcoholic Fatty Liver Disease. Front Endocrinol (Lausanne) 10: 611

Magne F, Gotteland M, Gauthier L, Zazueta A, Pesoa S, Navarrete P, Balamurugan R (2020) The Firmicutes/Bacteroidetes Ratio: A Relevant Marker of Gut Dysbiosis in Obese Patients? Nutrients 12: 1474

Munukka E, Rintala A, Toivonen R, Nylund M, Yang B, Takanen A, Hänninen A, Vuopio J, Huovinen P, Jalkanen S et al (2017) Faecalibacterium prausnitzii treatment improves hepatic health and reduces adipose tissue inflammation in high-fat fed mice. ISME J 11: 1667-1679

Segain JP, de la Blétière DR, Bourreille A, Leray V, Gervois N, Rosales C, Ferrier L, Bonnet C, Blottière HM, Galmiche JP (2000) Butyrate inhibits inflammatory responses through NFκB inhibition: implications for Crohn's disease. Gut 47: 397

Tamanai-Shacoori Z, Smida I, Bousarghin L, Loreal O, Meuric V, Fong SB, Bonnaure-Mallet M, Jolivet-Gougeon A (2017) Roseburia spp.: a marker of health? Future Microbiology 12: 157-170

10. The underlying molecular mechanism associated with the the reduced hepatic fat and inflammation in NAFLD patients and key player sin host-microbiome interactions should be underlined better and presented also in a graphical manner.

Response: We presented our main findings in the figure shown below. We now have added this figure as new Figure 8.

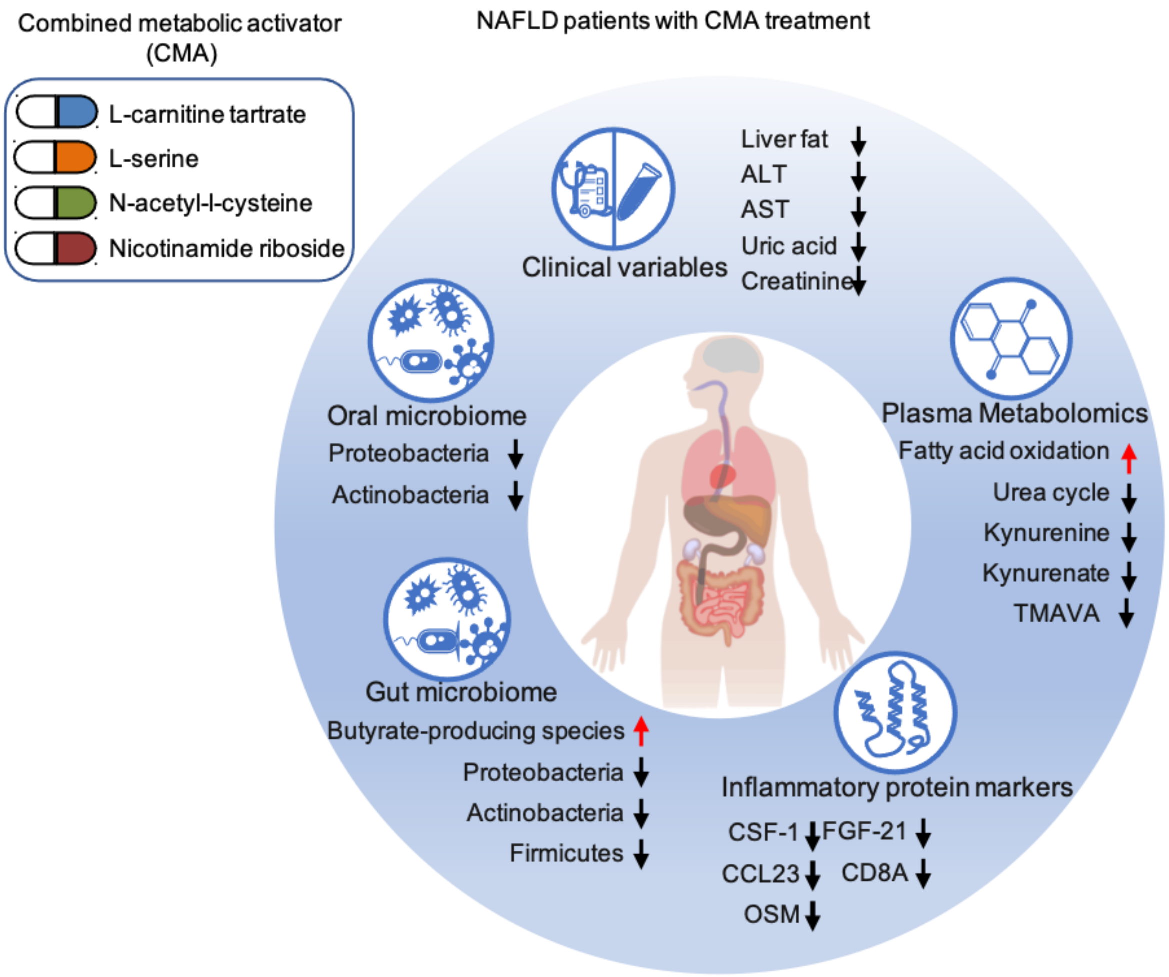
Combined metabolic activator (CMA)
L-carnitine tartrate
L-serine
N-acetyl-l-cysteine
Nicotinamide riboside
NAFLD patients with CMA treatment
Clinical variables
Liver fat
ALT
AST
Uric acid
Creatinine
Oral microbiome
Proteobacteria
Actinobacteria
Plasma Metabolomics
Fatty acid oxidation
Urea cycle
Kynurenine
Kynurenate
TMAVA
Gut microbiome
Butyrate-producing species
Proteobacteria
Actinobacteria
Firmicutes
Inflammatory protein markers
CSF-1
FGF-21
CCL23
CD8A
OSM

Thank you for sending us your revised manuscript. We have now heard back from the two reviewers who were asked to evaluate your study. As you will see the reviewers are overall satisfied with the modifications made and think that the study is now suitable for publication.

Before we can formally accept your manuscript, we would ask you to address the following editorial-level issues.

REFEREE REPORTS

Reviewer #1:

The authors have included all the suggestions in their revised manuscript and have clearly mentioned the limitations of the study. I am satisfied with the responses and the revised version of the manuscript.

Reviewer #3:

The authors have addressed majority of drawbacks that were present in the first reviewers report. The study remains small in size but a follow-up is planned based on authors' statement. The discussion is now more concise and underlines the novel findings in the light of the study drawbacks. Even if parts of the result session remain bulky (the microbiome part is still difficult to read), I suggest that the paper is accepted for publication. There is a major improvement from the first version and the positive effects of CMA are now described in a more clear manner.

The authors have made all requested editorial changes.

Thank you again for sending us your revised manuscript. We are now satisfied with the modifications made and I am pleased to inform you that your paper has been accepted for publication.

EMBO PRESS

YOU MUST COMPLETE ALL CELLS WITH A PINK BACKGROUND ➔

PLEASE NOTE THAT THIS CHECKLIST WILL BE PUBLISHED ALONGSIDE YOUR PAPER

Corresponding Author Name: ADIL MARDINOGLU
Journal Submitted to: MOLECULAR SYSTEMS BIOLOGY
Manuscript Number: MSB-2021-10459

USEFUL LINKS FOR COMPLETING THIS FORM

http://www.antibodypedia.com
http://1degreebio.org
http://www.equator-network.org/reporting-guidelines/improving-bioscience-research-repor
http://grants.nih.gov/grants/olaw/olaw.htm
http://www.mrc.ac.uk/Ourresearch/Ethicsresearchguidance/Useofanimals/index.htm
http://ClinicalTrials.gov
http://www.consort-statement.org
http://www.consort-statement.org/checklists/view/32-consort/66-title
http://www.equator-network.org/reporting-guidelines/reporting-recommendations-for-tumo
http://datadryad.org
http://figshare.com
http://www.ncbi.nlm.nih.gov/gap
http://www.ebi.ac.uk/ega
http://biomodels.net/
http://biomodels.net/miriam/
http://jjj.biochem.sun.ac.za
https://osp.od.nih.gov/biosafety-biosecurity-and-emerging-biotechnology/
http://www.selectagents.gov/

Reporting Checklist For Life Sciences Articles (Rev. June 2017)

This checklist is used to ensure good reporting standards and to improve the reproducibility of published results. These guidelines are consistent with the Principles and Guidelines for Reporting Preclinical Research issued by the NIH in 2014. Please follow the journal's authorship guidelines in preparing your manuscript.

A- Figures
1. Data
The data shown in figures should satisfy the following conditions:

- ➔ the data were obtained and processed according to the field's best practice and are presented to reflect the results of the experiments in an accurate and unbiased manner.
- ➔ figure panels include only data points, measurements or observations that can be compared to each other in a scientifically meaningful way.
- ➔ graphs include clearly labeled error bars for independent experiments and sample sizes. Unless justified, error bars should not be shown for technical replicates.
- ➔ if n< 5, the individual data points from each experiment should be plotted and any statistical test employed should be justified
- ➔ Source Data should be included to report the data underlying graphs. Please follow the guidelines set out in the author ship guidelines on Data Presentation.

2. Captions

Each figure caption should contain the following information, for each panel where they are relevant:

- ➔ a specification of the experimental system investigated (eg cell line, species name).
- ➔ the assay(s) and method(s) used to carry out the reported observations and measurements
- ➔ an explicit mention of the biological and chemical entity(ies) that are being measured.
- ➔ an explicit mention of the biological and chemical entity(ies) that are altered/varied/perturbed in a controlled manner.

- ➔ the exact sample size (n) for each experimental group/condition, given as a number, not a range;
- ➔ a description of the sample collection allowing the reader to understand whether the samples represent technical or biological replicates (including how many animals, litters, cultures, etc.).
- ➔ a statement of how many times the experiment shown was independently replicated in the laboratory.
- ➔ definitions of statistical methods and measures:
  - common tests, such as t-test (please specify whether paired vs. unpaired), simple χ2 tests, Wilcoxon and Mann-Whitney tests, can be unambiguously identified by name only, but more complex techniques should be described in the methods section;
  - are tests one-sided or two-sided?
  - are there adjustments for multiple comparisons?
  - exact statistical test results, e.g., P values = x but not P values < x;
  - definition of 'center values' as median or average;
  - definition of error bars as s.d. or s.e.m.

Any descriptions too long for the figure legend should be included in the methods section and/or with the source data.

In the pink boxes below, please ensure that the answers to the following questions are reported in the manuscript itself. Every question should be answered. If the question is not relevant to your research, please write NA (non applicable). We encourage you to include a specific subsection in the methods section for statistics, reagents, animal models and human subjects.

B- Statistics and general methods

Please fill out these boxes ➔ (Do not worry if you cannot see all your text once you press return)

1.a. How was the sample size chosen to ensure adequate power to detect a pre-specified effect size?	We included 31 NAFLD pateints in our statistical analysis, including 20 samples of NAFLD patients and 11 samples of placebo group. Since the sample size of each group is higher than 10, the statistical power should be enough.
1.b. For animal studies, include a statement about sample size estimate even if no statistical methods were used.	NA
2. Describe inclusion/exclusion criteria if samples or animals were excluded from the analysis. Were the criteria pre-established?	The sample pair with missing value was removed in a pairwise comparison.
3. Were any steps taken to minimize the effects of subjective bias when allocating animals/samples to treatment (e.g. randomization procedure)? If yes, please describe.	Yes, patient information (patient number, date of birth, initials) was entered into the web-based randomization system, and the randomization codes were entered into the electronic case report form.
For animal studies, include a statement about randomization even if no randomization was used.	NA
4.a. Were any steps taken to minimize the effects of subjective bias during group allocation or/and when assessing results (e.g. blinding of the investigator)? If yes please describe.	Yes, the randomization procedure is blind for the investigator.
4.b. For animal studies, include a statement about blinding even if no blinding was done	NA
5. For every figure, are statistical tests justified as appropriate?	Yes, we have described the test and the significance level we used in the figure legends. Moreover, the complete results of statistical analysis can be found in the corresponding supplementary files.
Do the data meet the assumptions of the tests (e.g., normal distribution)? Describe any methods used to assess it.	Yes, we followed the data transformation based on the test requirement. For example, limma requires a log transformation before calculation when we compared the differnece across time points within each group. In addition, we used one-way ANOVA to test the difference between different groups, it is non-parametric without the requirement for specific distribution.
Is there an estimate of variation within each group of data?	We used paired analysis when we compared the difference across different time points, so this is not calculated.

Is the variance similar between the groups that are being statistically compared?	We used one-way ANOVA when we compared the difference between different groups, which includes the consideration of the variance similarity between groups.

C- Reagents

6. To show that antibodies were profiled for use in the system under study (assay and species), provide a citation, catalog number and/or clone number, supplementary information or reference to an antibody validation profile. e.g., Antibodypedia (see link list at top right), 1DegreeBio (see link list at top right).	NA
7. Identify the source of cell lines and report if they were recently authenticated (e.g., by STR profiling) and tested for mycoplasma contamination.	NA

* for all hyperlinks, please see the table at the top right of the document

D- Animal Models

8. Report species, strain, gender, age of animals and genetic modification status where applicable. Please detail housing and husbandry conditions and the source of animals.	NA
9. For experiments involving live vertebrates, include a statement of compliance with ethical regulations and identify the committee(s) approving the experiments.	NA
10. We recommend consulting the ARRIVE guidelines (see link list at top right) (PLoS Biol. 8(6), e1000412, 2010) to ensure that other relevant aspects of animal studies are adequately reported. See author guidelines, under 'Reporting Guidelines'. See also: NIH (see link list at top right) and MRC (see link list at top right) recommendations. Please confirm compliance.	NA

E- Human Subjects

11. Identify the committee(s) approving the study protocol.	The study was approved by the ethics committee at Koç University (Decision No: 2018.351.IRB1.043).
12. Include a statement confirming that informed consent was obtained from all subjects and that the experiments conformed to the principles set out in the WMA Declaration of Helsinki and the Department of Health and Human Services Belmont Report.	The trial was conducted following Good Clinical Practice guidelines and the principles of the Declaration of Helsinki. An independent external data monitoring committee oversaw the safety of the participants and the risk-benefit analysis. Written informed consent was obtained from all participants before trial-related procedures were initiated.
13. For publication of patient photos, include a statement confirming that consent to publish was obtained.	No photos taken during the study
14. Report any restrictions on the availability (and/or on the use) of human data or samples.	No restrictions on the availabilty of the samples
15. Report the clinical trial registration number (at ClinicalTrials.gov or equivalent), where applicable.	The study was registered at https://eudract.ema.europa.eu/ with the accession number EudraCT_2018-000894-59 and at https://clinicaltrials.gov/ with the accession number NCT04330326.
16. For phase II and III randomized controlled trials, please refer to the CONSORT flow diagram (see link list at top right) and submit the CONSORT checklist (see link list at top right) with your submission. See author guidelines, under 'Reporting Guidelines'. Please confirm you have submitted this list.	List submitted
17. For tumor marker prognostic studies, we recommend that you follow the REMARK reporting guidelines (see link list at top right). See author guidelines, under 'Reporting Guidelines'. Please confirm you have followed these guidelines.	NA

F- Data Accessibility

18: Provide a "Data Availability" section at the end of the Materials & Methods, listing the accession codes for data generated in this study and deposited in a public database (e.g. RNA-Seq data: Gene Expression Omnibus GSE39462, Proteomics data: PRIDE PXD000208 etc.) Please refer to our author guidelines for 'Data Deposition'.<br><br>Data deposition in a public repository is mandatory for:<br>a. Protein, DNA and RNA sequences<br>b. Macromolecular structures<br>c. Crystallographic data for small molecules<br>d. Functional genomics data<br>e. Proteomics and molecular interactions	All data included in Expanded View Datasets
19. Deposition is strongly recommended for any datasets that are central and integral to the study; please consider the journal's data policy. If no structured public repository exists for a given data type, we encourage the provision of datasets in the manuscript as a Supplementary Document (see author guidelines under 'Expanded View' or in unstructured repositories such as Dryad (see link list at top right) or Figshare (see link list at top right).	All data included in Expanded View Datasets
20. Access to human clinical and genomic datasets should be provided with as few restrictions as possible while respecting ethical obligations to the patients and relevant medical and legal issues. If practically possible and compatible with the individual consent agreement used in the study, such data should be deposited in one of the major public access-controlled repositories such as dbGAP (see link list at top right) or EGA (see link list at top right).	NA
21. Computational models that are central and integral to a study should be shared without restrictions and provided in a machine-readable form. The relevant accession numbers or links should be provided. When possible, standardized format (SBML, CellML) should be used instead of scripts (e.g. MATLAB). Authors are strongly encouraged to follow the MIRIAM guidelines (see link list at top right) and deposit their model in a public database such as Biomodels (see link list at top right) or JWS Online (see link list at top right). If computer source code is provided with the paper, it should be deposited in a public repository or included in supplementary information.	NA

G- Dual use research of concern

22. Could your study fall under dual use research restrictions? Please check biosecurity documents (see link list at top right) and list of select agents and toxins (APHIS/CDC) (see link list at top right). According to our biosecurity guidelines, provide a statement only if it could.	NA
